# Complete abdominal wound and anastomotic leak with diffuse peritonitis closure achieved by an abdominal vacuum sealing drainage in a critical ill patient: a case report

**DOI:** 10.1186/s12893-018-0375-6

**Published:** 2018-06-15

**Authors:** Yusuke Fujii, Yoshitsugu Tajima, Shunsuke Kaji, Takashi Kishi, Yoshiko Miyazaki, Takahito Taniura, Noriyuki Hirahara

**Affiliations:** Department of Digestive and General Surgery, Shimane University Faculty of Medicine, 89-1, Enyacho, Izumo, Shimane 693-8501 Japan

**Keywords:** Anastomotic leakage, Peritonitis, Wound dehiscence, Negative pressure wound therapy

## Abstract

**Background:**

Negative pressure wound therapy (NPWT) is a widely accepted technique to treat local infectious wounds of the skin, subcutaneous tissue, fascia, or muscle. Recently, several reports describing the efficacy of NPWT for various types of fistulas and anastomotic leaks have been published. We herein describe a patient with an open abdominal wound due to colonic anastomotic leakage and diffuse peritonitis, in whom abdominal vacuum sealing (AVS) as a modified NPWT was useful for the management of this complex wound.

**Case presentation:**

A 32-year-old man was admitted to our hospital with late presenting traumatic diaphragmatic hernia and strangulated ileum complicated by necrosis of the ileum and transverse colon. He had a history of cervical spinal cord injury due to suicide attempt 14 years earlier and, as a result of cervical spinal cord injury, he was paralyzed in the lower body. The patient underwent an urgent hernia repair and bowel resection. Postoperatively, he developed severe septic shock. On postoperative day (POD) 6, wound dehiscence due to colonic anastomotic leakage with diffuse peritonitis was diagnosed, but he was unable to undergo re-operation because of refractory severe septic shock combined with neurogenic shock due to the cervical cord injury. The patient was treated with AVS therapy. He gradually recovered from septic shock, and the anastomotic leakage healed after a 2-month period. The wound dehiscence was also reduced. The patient resumed oral intake on POD 112 and was discharged on POD 190.

**Conclusions:**

Although surgical repair would be the best method for the treatment of diffuse peritonitis due to gastrointestinal perforation or anastomotic leakage, our case suggests that AVS with ‘conventional’ drainage is a treatment of choice for open abdominal wounds even in the presence of diffuse peritonitis caused by intestinal anastomotic leakage, especially in patients with poor general medical condition.

## Background

Negative pressure wound therapy (NPWT) was first reported in 1997 as delivered by vacuum-assisted closure [1, 2]. It is a useful therapeutic strategy and has become the most common treatment tool for complex wounds. The controlled negative pressure of NPWT results in a decrease in bacterial colonization, tissue edema, and wound tension, as well as an increase in blood flow [1]. NPWT promotes wound granulation, and has been used for chronic, traumatic, and open abdominal wounds. Recently, several reports have been published on the efficacy of NPWT for various types of enteric fistulas and anastomotic leaks [3–6]. However, there have been no reports regarding the use of NPWT for open abdominal wounds with diffuse peritonitis secondary to anastomotic leakage or fistula. In patients with postoperative diffuse peritonitis due to anastomotic leakage and subsequent infectious open wound, surgical repair would be the best management to save the patient’s life and control wound infection; however, some patients are unable to undergo surgery due to their deteriorated general condition. In such cases, an appropriate drainage is indispensable but that alone insufficient for saving the patient’s life and healing the anastomotic leakage. Herein we describe an inoperable case of diffuse peritonitis and open abdominal wound caused by colonic anastomotic leakage in a patient with poor general condition, in whom an abdominal vacuum sealing (AVS) was useful for healing diffuse peritonitis, the complex wound, and even the anastomotic leakage.

## Case presentation

**Fig. 1:**
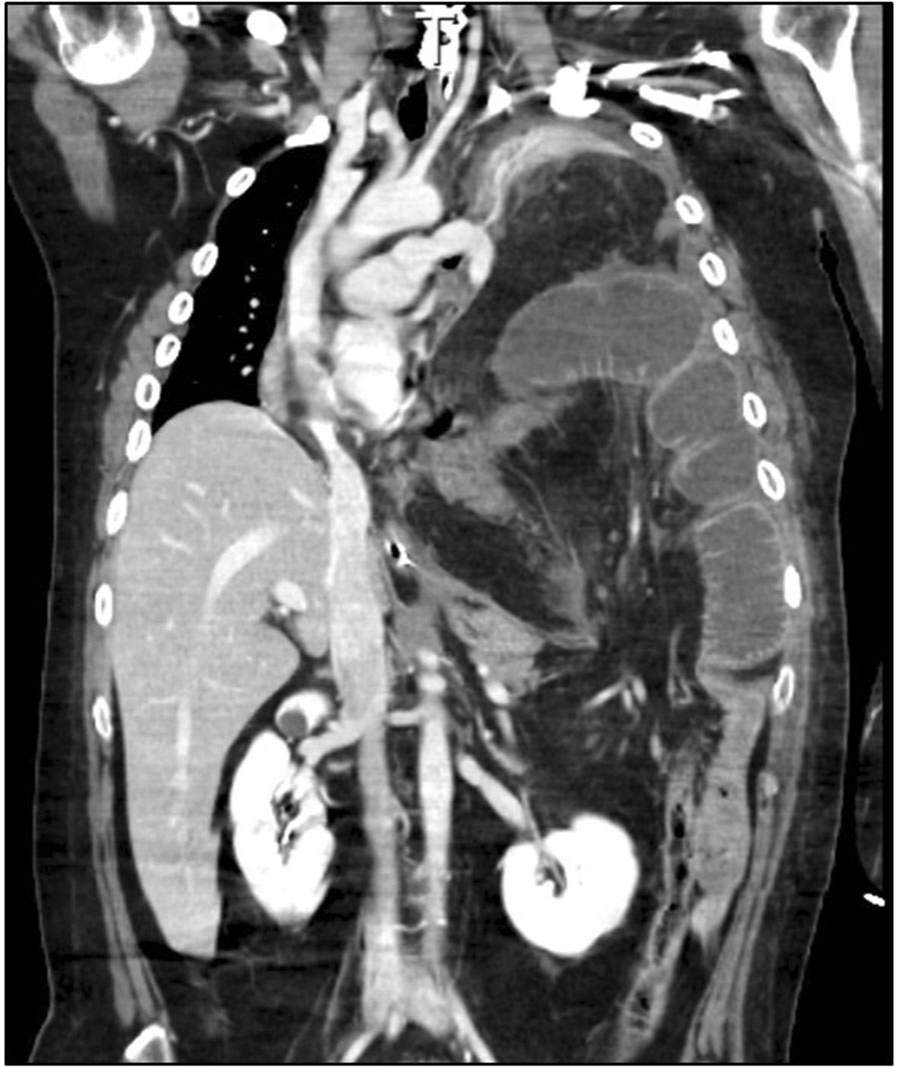
Contrast-enhanced computed tomography (CT). Images demonstrated the presence of a large diaphragmatic hernia, which allowed the small intestine, transverse colon, and omentum to herniate into the left chest. Portions of these herniated organs showed decreased blood flow. Collapse of the left lung and marked mediastinal shift to the right were apparent

**Fig. 2:**
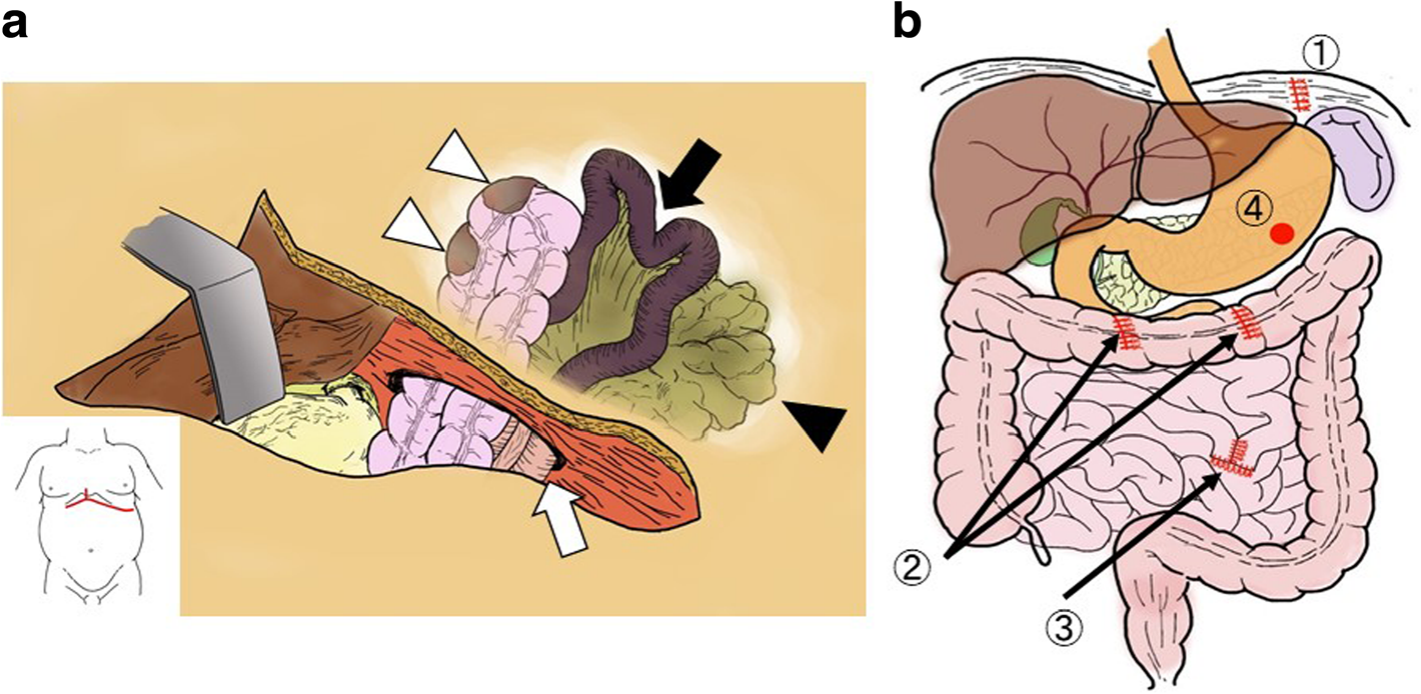
Schemata of the operative findings. **a** Diaphragmatic hernia with strangulated ileus: scattered areas of necrosis in the small intestine (black arrow), transverse colon (white arrowheads), and omentum (black arrowhead) were evident. **b** The orifice of the diaphragmatic hernia was closed with interrupted sutures (1). The necrotic portions of the transverse colon (2) and small intestine (3) were resected and repaired. A gastrostomy was performed (4)

**Fig. 3:**
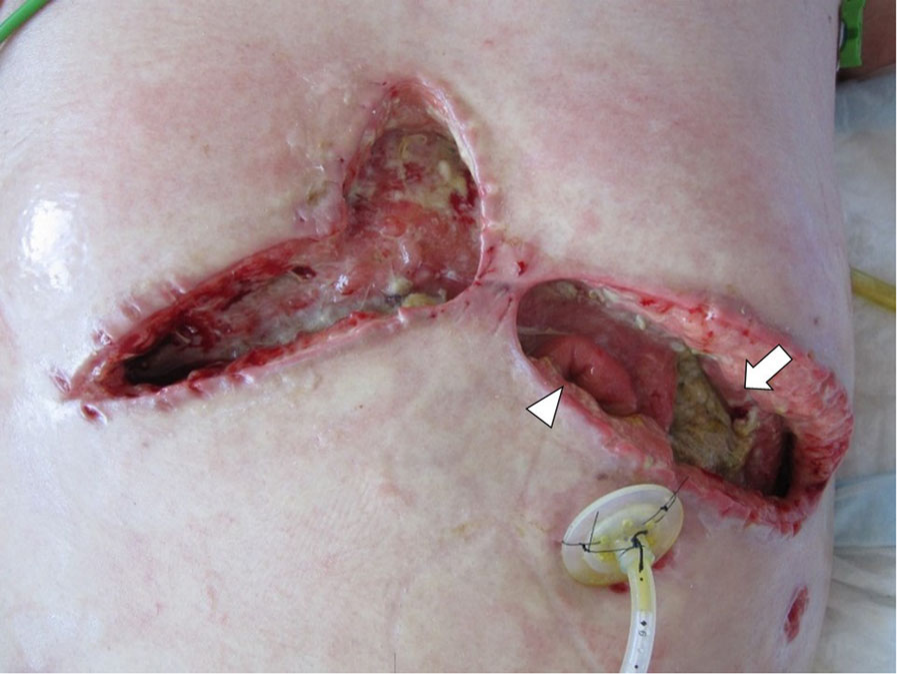
The open abdominal wound on postoperative day 6. Wound dehiscence with collapse of the colonic anastomosis (arrow) and gastrostomy (arrowhead) was recognized

**Fig. 4:**
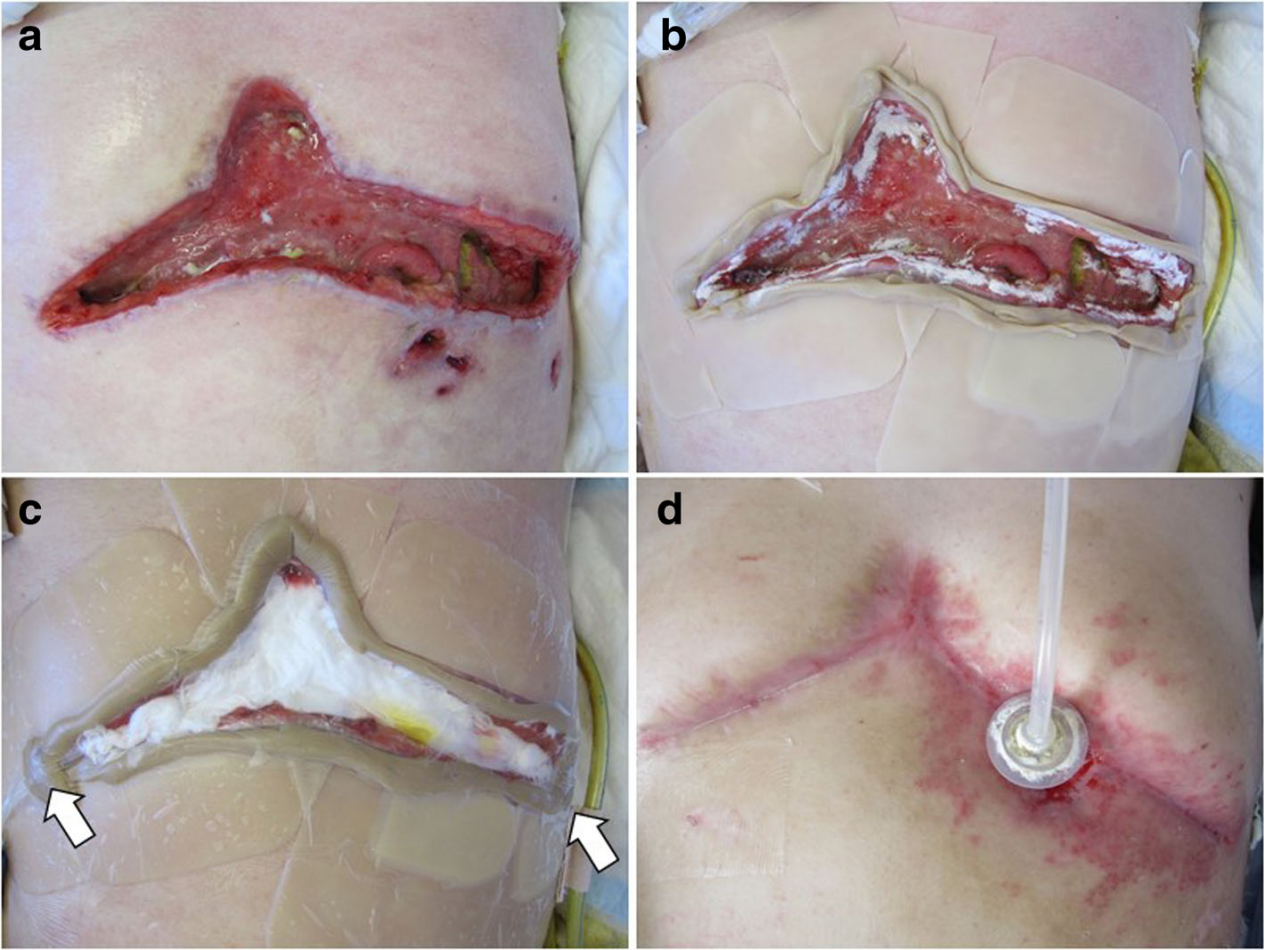
Procedure for abdominal vacuum sealing. **a** The open wound and peritoneal cavity were washed with saline and necrotic and/or contaminated tissues were debrided. **b** Wound dressing materials were placed along the abdominal wound to protect healthy skin around the wound. **c** Two drainage tubes (arrows) with multiple side holes, up to 30 cm from the tip, were placed in the abdominal cavity through the open abdomen to drain the gastrointestinal contents. Gastrointestinal contents were suctioned through the drainage tubes with negative pressure. **d** The gastric fistula remained, but other gastrointestinal anastomotic leakages were healed. The gastrostomy balloon was inserted in the gastric fistula

A 32-year-old man was admitted to our hospital with appetite loss. He had a history of traumatic transverse cervical spinal cord injury at the C5 level due to suicide attempt at the age of 18. As a result of cervical spinal cord injury, he was paralyzed in the lower body. Contrast-enhanced computed tomography (CT) revealed a late-onset traumatic diaphragmatic hernia with strangulated ileum (Fig. 1). The small intestine, transverse colon, and omentum were displaced into the left thoracic cavity, and some portions of these organs showed a decrease in blood flow. Left lung collapse and a compressed right lung with mediastinal shift were evident. The patient underwent emergency surgery. After replacing the incarcerated organs to their original positions, scattered areas of necrosis were identified in the small intestine, transverse colon, and omentum (Fig. 2a). By using interrupted sutures with non-absorbable 1–0 monofilament, the diaphragmatic orifice was closed. Wedge resection with primary closure was performed for the colonic necrosis in two places. Partial resection, 45 cm long, with end-to-end anastomosis was performed for the small intestine. The necrotic omentum was removed (Fig. 2b). In addition, a gastrostomy tube was placed since delayed initiation of oral intake was expected. The patient developed severe septic shock postoperatively. Treatment-resistant critical hypotension with non-compensatory tachycardia developed, likely due to parasympathetic nervous system damage related to the cervical spinal cord injury. On postoperative days (POD) 3 and 6, cardiac arrest occurred. Fortunately, he was rescued by cardiopulmonary resuscitation with administration of large doses of vasopressin and catecholamine. However, peripheral vasoconstriction, increased intra-abdominal pressure, and ischemia of the gastrointestinal tract developed, which resulted in colonic anastomotic leakage with diffuse peritonitis, abdominal wound dehiscence, and collapse of gastrostomy on POD 6 (Fig. 3). The patient was unable to undergo surgical repair because of his poor general condition with continuing severe septic and neurogenic shock. Therefore, he underwent AVS through the open abdominal wound and it was the first procedure at the intensive care unit. The procedure of AVS was as follows: 1. the open wound and peritoneal cavity were rinsed with normal saline and necrotic and/or contaminated tissues were debrided (Fig. 4a); 2. wound dressing materials (DUOACTIVE® ConvaTec, New Jersey, USA) for protecting healthy skin around the open wound were patched along the abdominal wound in piecemeal fashion so as to adjust dressing materials to the complicated shape of the wound (Fig. 4b); 3. two drainage tubes with multiple side holes, up to 30 cm from the tip, were placed in the abdominal cavity through the open abdomen and the enteric contents were suctioned through the drainage tubes using a Continuous Suction Unit MERA Sacuum (Senko Medical Instrument Manufacturing CO, Tokyo, Japan) set to 50–75 mmHg continuous negative pressure; and 4) the entire wound was filled with saline-moistened gauzes and covered with polyurethane drape (Fig. 4c). The colonic anastomotic leakage showed gradual healing over the course of 2 months, followed by contraction and closure of wound dehiscence (Fig. 4d). Because the gastric fistula remained, a gastrostomy balloon catheter was placed through the gastric fistula. The patient resumed oral intake on POD 112 and left the hospital on POD 190 with the gastrostomy balloon catheter and without incisional hernia.

## Discussion and conclusions

Enterocutaneous fistulas are uncommon surgical problems, but they pose challenges to their successful management and healing due to underlying serious pathological conditions, such as severe malnutrition, electrolyte imbalances, and sepsis, with a mortality rate ranging from 6 to 33% [7, 8]. Enterocutaneous fistulas can be categorized as simple or complex. Simple fistulas are short with a direct tract, and are not associated with an abscess or the involvement of other organs. Complex fistulas are categorized into two types: type 1 fistulas that are associated with an abscess or multiple organ involvement, and type 2 fistulas that open into the base of a disrupted wound, often termed an enteroatmospheric fistula [9]. Enteroatmospheric fistula is defined as “an exposed fistula occurring in the midst of an open abdomen with no overlying soft tissue” [9]. Complex fistulas have a lower rate of spontaneous closure with higher morbidity and mortality [10].

NPWT is a useful approach for wound closure by second intention [1, 2]. In the past, NPWT was contraindicated in the management of patients with gastrointestinal fistula because it appeared to cause delay in wound closure and even internal organ damage [11]. Recently, several reports have been published concerning the efficacy of NPWT in the treatment of various types of fistulas [3–6]. Regarding the management of patients with enteroatmospheric fistula, a few reports have demonstrated the efficacy of NPWT [6]. However, there has been no report of NPWT therapy for wound dehiscence with diffuse peritonitis due to an enteric anastomotic leakage or gastrointestinal perforation. To the best of our knowledge, our report presents the first successful case of AVS therapy for an open abdominal wound with diffuse peritonitis related to enteric anastomotic leakage.

In patients with enteroatmospheric fistula, evidence suggests that extensive wound care such as NPWT is effective and that nutritional support can minimize the delay of recovery and improve patient outcomes [12]. Isolation of the gastrointestinal contents is one of the most important factors for preventing the spread of infections and for promoting wound healing. It has been demonstrated that NPWT under low intestinal pressure is effective for the management of fistulas [6, 7, 13]. In our patient, total parenteral nutrition was utilized to decrease the enteral pressure as well as the intestinal fluid secretion and to reduce the contamination of the wound and abdominal cavity with intestinal fluids.

Surgical management is generally considered the best method to control diffuse peritonitis with gastrointestinal perforation or anastomotic leakage. However, in patients with exceedingly poor general condition, drainage remains the only treatment option. NPWT for the abdominal cavity was effective in stimulating granulation tissue formation, covering both the gastrointestinal wall and wound bed, and promoting wound healing in our patient. In our AVS procedure, drainage tubes with multiple side holes, up to 30 cm from the tip, were placed in the abdominal cavity through the open abdomen to drain the gastrointestinal fluids and, in addition, the wound was covered with saline-moistened gauzes and wrapped with polyurethane drape. These were the techniques used in our AVS management, which allowed for effective peritoneal drainage and also protected the open wound from contamination with enteric contents. In addition, the gauze dressings should be changed frequently, every 2–3 days, even without macroscopic pollution [14].

In conclusion, AVS can be considered a treatment of choice for complex wounds, including an enteroatmospheric fistula, especially in patients with poor general condition.

## Abbreviations

AVS: Abdominal vacuum sealing
CT: Computed tomography
NPWT: Negative pressure wound therapy
POD: Postoperative days

## References

[1] Morykwas MJ, Argenta LC, Shelton-Brown EI (1997). Vacuum-assisted closure: a new method for wound control and treatment: animal studies and basic foundation. Ann Plast Surg.

[2] Argenta LC, Morykwas MJ (1997). Vacuum-assisted closure: a new method for wound control and treatment: clinical experience. Ann Plast Surg.

[3] Navsaria PH, Bunting M, Omoshoro-Jones J (2003). Temporary closure of open abdomen wounds by the modified sandwich-vacuum pack technique. Br J Surg.

[4] Medeirois AC, Aires-Neto T, Marchini JS (2004). Treatment of post-operative enterocutaneous fistulas by high pressure vacuum with a normal oral diet. Dig Surg.

[5] Goverman J, Yelon JA, Platz JJ (2006). The “fistula VAC” atechnique for management of enterocutaneous fistulae arising within the open abdomen: report of 5 cases. J Trauma.

[6] Shiki F, Norikatsu M, Masayuki O (2015). Use of vacuum-assisted closure in management of open abdominal wound with multiple enterocutaneous fistulae during chemotherapy: a case report. Int J Surg Case Report.

[7] Rahbour G, Siddiqui MR, Ullah MR (2012). A meta-analysis of outcomes following use of somatostatin and its analogues for the management of enterocutaneous fistulas. Ann Surg.

[8] Evenson AR, Fischer JE (2006). Current management of enterocutaneous fistula. J Gastrointest Surg.

[9] Shecter W (2014). Principles of management of enteric fistulas.

[10] Wong WD, Buie WD (1993). Management of intestinal fistulas. Intestinal stomas, quality medical publishing, Missouri.

[11] Banasiewicz T, Borajsza-Wysocki M, Meissner W (2011). Vacuum-assisted closure therapy in patients with large postoperative wounds complicated by multiple fistulas. Wideochir Inne Tech Maloinwazyjne.

[12] Wainstein DE, Tungler V, Ravazzola C (2011). Management of external small bowel fistulae: challenges and controversies confronting the general surgeon. Int J Surg.

[13] Ozer MT, Sinan H, Zeybek N (2014). A simple novel technique for enteroatmospheric fistulae: silicone fistula plug. Int Wound J.

[14] Parathoner A, Klaus A, Muhlmann G (2010). Damage control with abdominal vacuum therapy (VAC) to manage perforated diverticulitis with advanced generalized peritonitis – a proof of concept. Int J Cororectal Dis.

